# Initiation of programmed cell death in the suspensor is predominantly regulated maternally in a tobacco hybrid

**DOI:** 10.1038/srep29467

**Published:** 2016-07-19

**Authors:** An Luo, Peng Zhao, Li-Yao Zhang, Meng-Xiang Sun

**Affiliations:** 1 College of Life Science, State Key Laboratory of Hybrid Rice, Wuhan University, Wuhan 430072, China; 2 College of Life Science, Yangtze University, Jingzhou, 434023, China

## Abstract

Maternal gene products deposited in the egg regulate early embryogenesis before activation of the embryonic genome in animals. While in higher plants, it is believed that genes of parental origin contribute to early embryogenesis. However, little is known regarding the particular processes in which genes of parental origin are involved during early embryogenesis. Previously, we found that the initiation of programmed cell death (PCD) in the suspensor of the embryo is regulated by the cystatin, NtCYS. Here, we confirmed that both parental transcripts contribute to PCD, but the relative expression level of the maternal *NtCYS* allele was much higher than that of the paternal allele in early embryos of tobacco interspecific hybrids. The expression level of the maternal *NtCYS* allele was decreased markedly, which was necessary for the initiation of PCD, while the paternal allele didn’t change. Interestingly, the pattern of PCD in the hybrid suspensor and the morphology of the hybrid suspensor were similar to those of the maternal parent. Our results suggest that NtCYS-mediated PCD initiation in the hybrid suspensor is likely controlled in a maternal dominant manner. This finding represents an example of the involvement of parental transcripts in a specific developmental event during early embryogenesis.

In higher plants, the zygote, derived from the fusion of an egg and a sperm cell, represents the initiation of embryogenesis. After asymmetric cell division, the zygote produces two daughter cells with distinct cell fates. The apical cell forms the embryo proper, which constitutes the majority of the mature embryo. The basal cell develops mainly into the suspensor, which supports embryonic development^1^,^2^,^3^,^4^. Although the suspensor, as a short-lived structure, is not required for the later stages of embryonic development and is ultimately degraded, it is essential for early embryogenesis, not only for fixing the embryo in the nourishing endosperm spatially, but also for nutrient and hormone transport to the embryo^5^,^6^.

Early embryonic development is mainly controlled maternally in animals^7^, whereas in higher plants, transcripts of both paternal and maternal origin are thought to contribute to zygote development and early embryogenesis^8^,^9^. However, little is known regarding the particular developmental processes regulated by parental transcripts and how they contribute to embryogenesis in interspecies tobacco hybrids. Recently, a predicted exopolygalacturonase gene, *NIMNA* (*NMA*), which can interfere with cell elongation and suspensor length in early embryogenesis, was found to have a paternal allele influence on the embryonic phenotype^10^. This suggests that different developmental processes may be regulated differentially by genes of paternal or maternal origin. Previously, we found that the initiation of programmed cell death (PCD) in the suspensor is regulated by a protease inhibitor, cystatin NtCYS, and the detailed process of PCD was investigated. In the early proembryo, the level of *NtCYS* expression remains high during suspensor formation. Once the suspensor has formed, however, *NtCYS* expression is reduced rapidly, while its substrate protease, NtCP14, is upregulated, thereby triggering PCD in the suspensor^11^. As the suspensor is a transient structure and the hybrid suspensor observed in our previous study showed obvious maternal characters, we suspected that suspensor cell fate is controlled maternally. Therefore, we aimed to determine whether the specific PCD process is regulated by uniparental transcripts and how it can be influenced by the parental alleles of *NtCYS*.

Based on the cross system in tobacco (Hamayan × SR1; the maternal parent is denoted first) successfully established in our laboratory^12^, we developed a new method to allow the production of reciprocal hybrids (SR1 × Hamayan), which allows investigation of the relative expression level of *NtCYS* alleles from the two parents in tobacco hybrids after reciprocal crosses during early embryogenesis. The time course and pattern of PCD in the suspensor during early embryogenesis were also recoded, and comparative analysis among the parents and their hybrids was performed using a cell death marker array. Our results indicated that the PCD process in the suspensor is regulated by both parental transcripts with maternal dominance.

## Results

### Selection of parental species for hybridization and subsequent analysis

*NtCYS* was shown to play a critical role in the initiation of PCD in the tobacco suspensor (*Nicotiana tabacum* var. SR1). We next determined whether this process is influenced by uniparental gene expression. For this purpose, different cultivars or species with distinguishable *NtCYS* alleles as parental material are required. Eleven cultivars of *N. tabacum* were selected to determine the nucleotide sequences of *NtCYS* alleles, but no single nucleotide polymorphisms (SNPs) were found in the *NtCYS* alleles between these cultivars and SR1 (Supplementary Table S1). Consequently, it is not possible to identify the parental origin of *NtCYS* transcripts in the hybrids based on nucleotide differences. Therefore, two tobacco species, *N. tabacum* var. SR1 and *Nicotiana rustica* var. Hamayan, were further selected for successful hybridization^12^ (Hamayan × SR1), and the reciprocal hybridization event (SR1 × Hamayan) was also performed successfully by pollinating on shortened styles (Supplementary Fig. S1). Embryogenesis of reciprocal hybrids occurred normally, as in their parental lines (Supplementary Fig. S2). In addition, nucleotide differences between Hamayan and SR1 were found and confirmed in the *NtCYS* alleles, which enabled detection of the parental contributions of *NtCYS* alleles in the reciprocal hybrids.

### Comparison of suspensor development between two tobacco species

**Figure 1:**
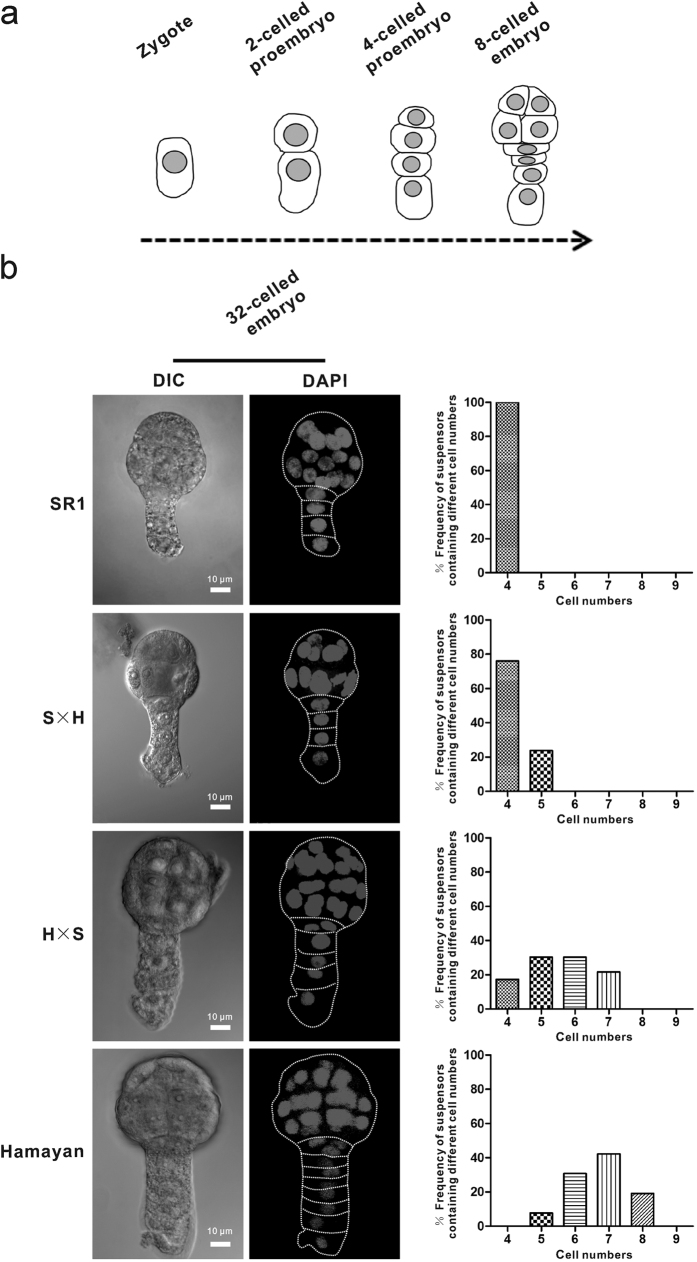
Early embryogenesis and the comparison of suspensor development among SR1, Hamayan and their reciprocal hybrids. (**a**) The embryo development from zygote to eight-celled embryo stage in SR1, Hamayan and their hybrids. (**b**) 32-celled embryos of SR1, hybrid (SR1 × Hamayan), hybrid (Hamayan × SR1) and Hamayan. The frequency of suspensors containing different cell numbers in SR1, hybrid (SR1 × Hamayan), hybrid (Hamayan × SR1) and Hamayan were analyzed respectively (n = 21–26).

In embryonic development of SR1, following zygotic cell division, the basal cell undergoes two successive divisions, giving rise to a four-celled suspensor at the eight-celled embryo stage. This process is similar to that seen in Hamayan and the reciprocal hybrids (Fig. 1a). However, there are differences in later suspensor development among the embryos of the four lines. The four-celled suspensor was maintained until the 32-celled embryo stage in SR1 (Fig. 1b). Prior to its degeneration, the cell number of the suspensor no longer increased. In contrast, the four-celled suspensor of Hamayan underwent divisions to form a five ~ eight-celled suspensor at the 32-celled embryo stage (Fig. 1b). Interestingly, the suspensor development of hybrids was highly similar to that of the maternal line with regard to both morphology and cell number (Fig. 1b).

### Construction of cDNA pools of two-celled proembryos and eight-celled embryos

**Figure 2:**
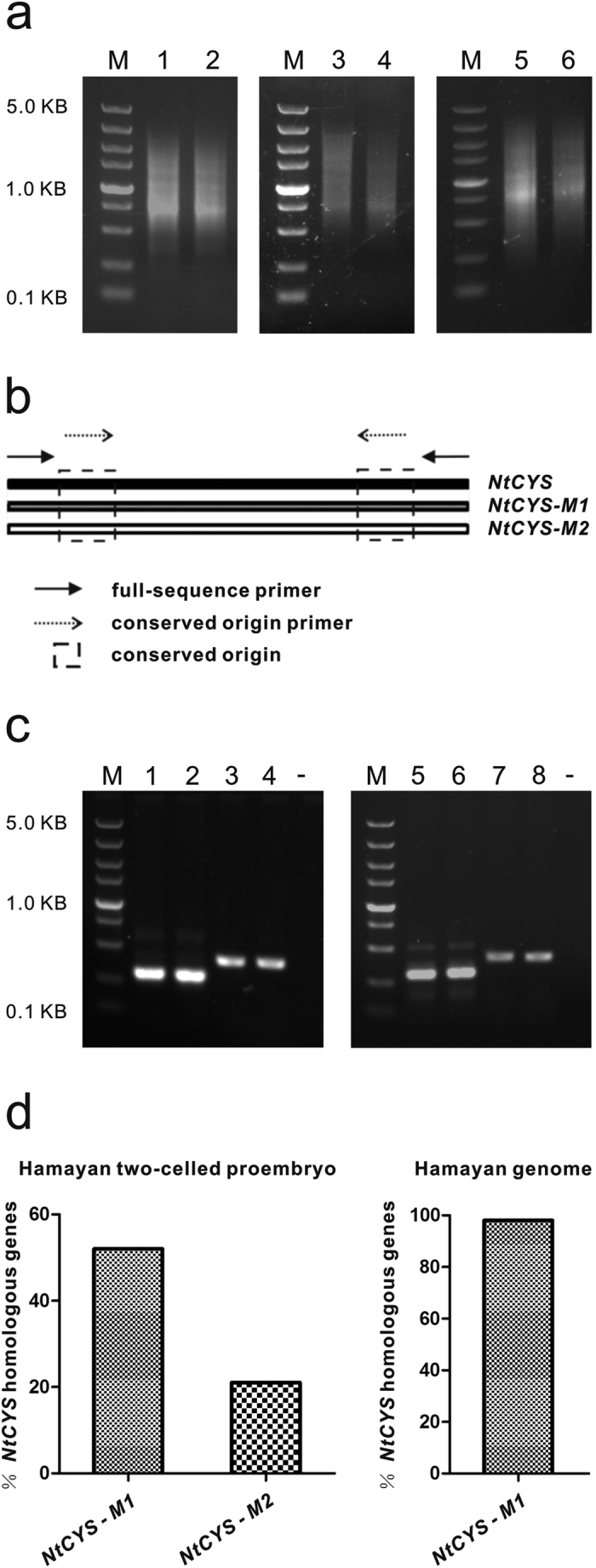
Construction of cDNA pools and detection of *NtCYS* homologous genes. (**a**) 1–6: cDNA pools of two-celled proembryo from Hamayan, two-celled proembryo from SR1, eight-celled hybrid embryo (SR1 × Hamayan), two-celled hybrid proembryo (SR1 × Hamayan), eight-celled hybrid embryo (Hamayan × SR1), two-celled hybrid proembryo (Hamayan × SR1); M: marker. (**b**) Diagram of primers’ location and conserved region in *NtCYS* homologous genes. Solid arrow: primer pair 1; dotted arrow: primer pair 2; dotted boxes: the conserved region. (**c**) Detection of *NtCYS* homologous genes. 1–2 Amplification of primer pair 2 in the two-celled proembryo cDNA pool from Hamayan and SR1; 3–4. Amplification of primer pair 1 in the two-celled proembryo cDNA pool from Hamayan and SR1; 5–6. Amplification of primer pair 2 in the genome of Hamayan and SR1; 7–8. Amplification of primer pair 1 in the genome of Hamayan and SR1; - negative control; M: marker. (**d**) The percentage of *NtCYS* homologous genes detected by primer pair 1 in Hamayan two-celled proembryos and Hamayan genome, n = 48 and 50 respectively.

A well-established method based on enzymatic maceration combined with brief dissection allowed us to isolate sufficient numbers of living two-celled proembryos and eight-celled embryos to construct cDNA pools. Embryos of SR1, Hamayan, and their reciprocal hybrids were isolated by a method described previously^12^,^13^,^14^. The mRNA from embryos was then isolated, and cDNA was synthesized and amplified. Each individual cDNA pool was then constructed successfully (Fig. 2a).

### Detection of the *NtCYS* allele in Hamayan

Previously, Zhao *et al*.^11^ confirmed that *NtCYS* in SR1 is only active during early embryogenesis and directly regulates suspensor PCD. To determine whether PCD in the suspensor mediated by *NtCYS* is regulated by uniparental transcripts in the reciprocal hybrids, we first confirmed whether the *NtCYS* allele was indeed expressed in Hamayan early embryos.

We designed primer pair 1 based on the *NtCYS* sequence in SR1. Because primer pair 1 should theoretically amplify the full-length sequence of *NtCYS* homologs (Fig. 2b), it was used to amplify the target gene in the two-celled proembryo cDNA pool from Hamayan. The PCR results showed that *NtCYS* homologs were expressed in Hamayan two-celled proembryos (Fig. 2c). A unique band was extracted and sequenced, and some homologs were found. Two genes with high frequencies of 52% and 21% were designated as *NtCYS-M1* and *NtCYS-M2*, respectively (Fig. 2d). Other homologous genes significantly fewer clones detected were ignored in our analyses. Bioinformatics analysis indicated that the two genes, *NtCYS-M1* and *NtCYS-M2*, showed 94% and 93% similarity to *NtCYS*, respectively. In addition, primer pair 1 was used to amplify the target gene in the Hamayan genome. A unique band was extracted and sequenced, and *NtCYS-M1* was found at a high frequency of 98% (Fig. 2c,d). Therefore, *NtCYS-M1* is likely the *NtCYS* allele in Hamayan.

### The relative expression levels of *NtCYS* alleles in reciprocal hybrids at the two-celled proembryo and eight-celled embryo stages

By sequence alignment, we found two conserved regions without any SNPs in *NtCYS*, *NtCYS-M1* and *NtCYS-M2*. Based on these sequences, we designed primer pair 2 (Fig. 2b). This primer pair ensures that PCR amplification will only be influenced by the abundance of the alleles expressed in hybrid embryos. Therefore, this allows determination and comparison of the relative expression levels of the *NtCYS* alleles in two-celled proembryos and eight-celled hybrid embryos.

**Figure 3:**
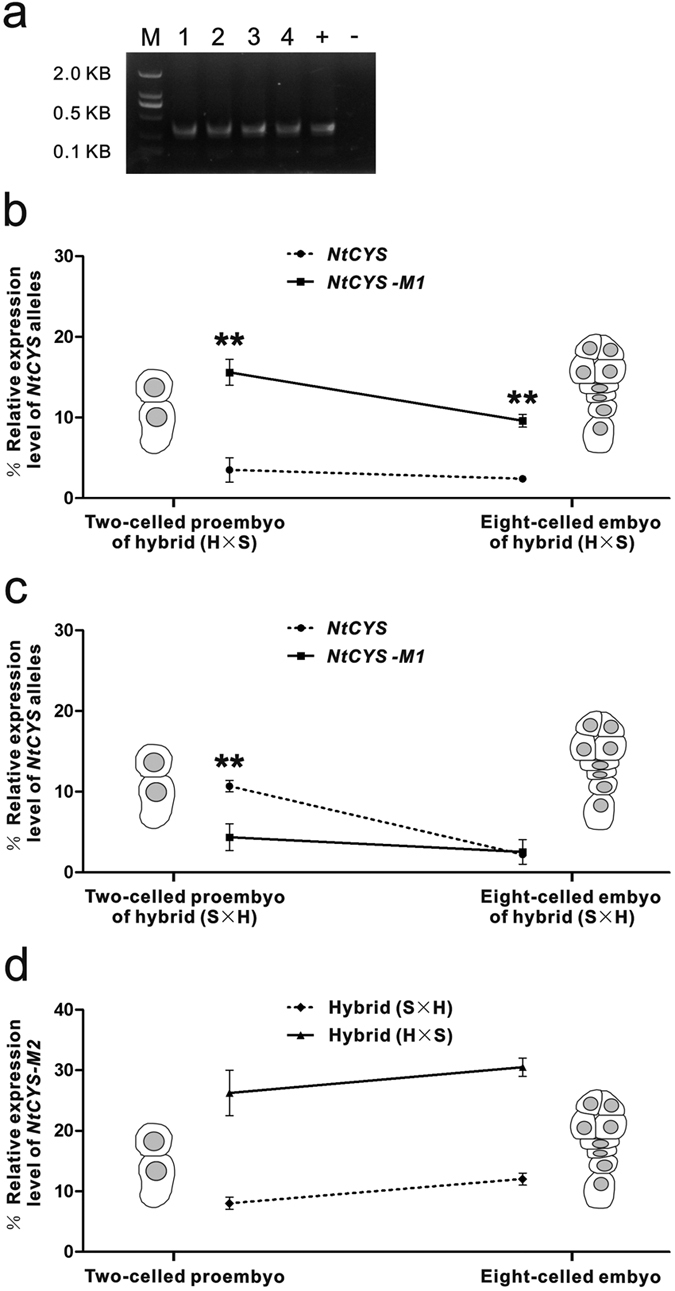
Relative expression levels of *NtCYS* alleles in the early embryogenesis of the hybrids. (**a**) Detection of *NtCYS* homologous genes. 1–4. primer pair 2 were amplified in the cDNA pools of eight-celled hybrid embryo (Hamayan × SR1), two-celled hybrid proembryo (Hamayan × SR1), eight-celled hybrid embryo (SR1 × Hamayan) and two-celled hybrid proembryo (SR1 × Hamayan), respectively; - indicates negative control; +indicates positive control. M: marker. (**b**) Comparative analysis of *NtCYS* and *NtCYS-M1* expression at two-celled proembryo and eight-celled embryo stages (Hamayan × SR1). Data represent the mean ± SE. Data were analyzed by Chi-square test (two-celled hybrid proembryo stage: χ2 = 0.000, **p < 0.01, n = 221, the percentage of *NtCYS* and *NtCYS-M1* was 16% and 3.5% respectively; eight-celled hybrid embryo stage: χ2 = 0.003, **p < 0.01, n = 210, the percentage of *NtCYS* and *NtCYS-M1* was 9.6% and 2.4%respectively). (**c**) Comparative analysis of *NtCYS* and *NtCYS-M1* expression at two-celled proembryo and eight-celled embryo stages (SR1 × Hamayan). Data represent the mean ± SE. Data were analyzed by Chi-square test (two-celled hybrid proembryo stage: χ2 = 0.006, **p < 0.01, n = 260, the percentage of *NtCYS* and *NtCYS-M1* was 11% and 4.4% respectively; eight-celled hybrid embryo stage: χ2 = 0.513, n = 396, the percentage of *NtCYS* and *NtCYS-M1* was 2.2% and 2.5% respectively). (**d**) Relative expression level of *NtCYS-M2* in two-celled proembryos and eight-celled embryos of the hybrids from reciprocal crosses. Data represent the mean ± SE, n = 210–396.

The actual amplification resulted in a unique band, which extracted and sequenced (Fig. 3a). In the hybrid (Hamayan × SR1, the maternal parent is denoted first), the relative expression level of *NtCYS-M1* was much higher than that of *NtCYS* during early embryogenesis. In addition, the relative expression level of *NtCYS-M1* decreased from the two-celled proembryo to the eight-celled embryo stage (from 16% to 9.6%), while the relative expression level of *NtCYS* showed little change and remained at a very low level (from 3.5% to 2.4%) (Fig. 3b).

The statistics clearly showed that the relative expression level of *NtCYS-M1* decreased after suspensor formation, but remained much higher than that of *NtCYS*. This was consistent with the expression pattern of *NtCYS* in SR1. As an inhibitor, *NtCYS* expression is gradually silenced after formation of the suspensor during PCD^11^. These results suggested that *NtCYS-M1* is the *NtCYS* allele in Hamayan. More importantly, this result indicated that the level of the *NtCYS-M1* transcript from the maternal genome is much higher than those of the alleles from the paternal genome, suggesting that PCD in the suspensor is mediated by *NtCYS* and involves both parental genomes and regulates early embryogenesis of the hybrid (Hamayan × SR1) in a maternal predominant manner.

Similarly, in the hybrid (SR1 × Hamayan), the relative expression level of *NtCYS-M1*, compared with *NtCYS*, was lower at the two-celled proembryo stage and was approximately equal at the eight-celled embryo stage. The relative expression level of *NtCYS* also decreased from the two-celled proembryo to the eight-celled embryo stage (from 11% to 2.2%), while the relative expression level of *NtCYS-M1* remained at a consistently low level during the same developmental process (from 4.4% to 2.5%) (Fig. 3c). These observations suggested that *NtCYS*-mediated PCD in the suspensor involves both parental genomes but is regulated in a maternal predominant manner during early embryogenesis in the hybrid (SR1 × Hamayan).

The relative expression levels of *NtCYS-M2* were also determined in two-celled hybrid proembryos and eight-celled hybrid embryos after reciprocal crosses. In contrast to *NtCYS-M1*, *NtCYS-M2* did not lose activity as the embryo developed. This suggested that *NtCYS-M2* is involved in certain processes other than suspensor PCD (Fig. 3d).

### Comparison of PCD initiation in the suspensor among SR1, Hamayan, and reciprocal hybrids during the early stages of embryonic development

**Figure 4:**
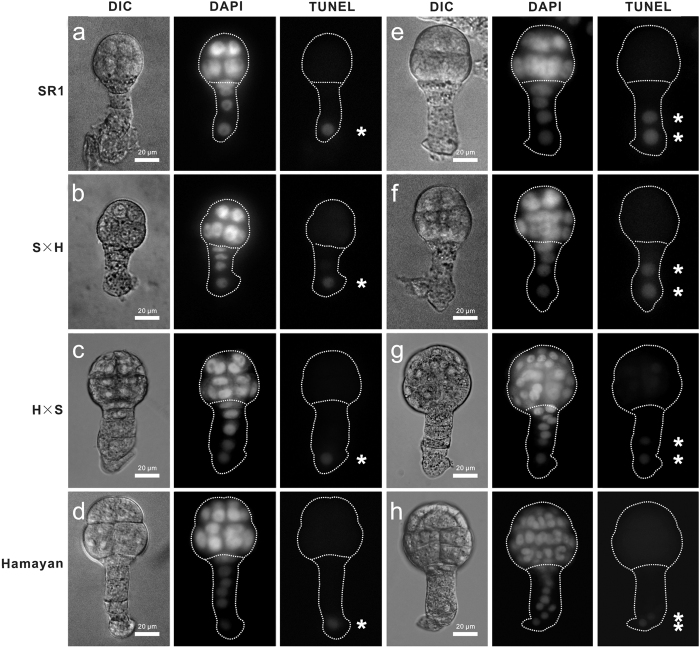
Dynamics of DNA fragmentation in the suspensors of SR1, Hamayan and their reciprocal hybrids at early stages. (**a**) Nuclear DNA fragmentation in the suspensor revealed by TUNEL at the 16-celled embryo stage in SR1. (**b**) Nuclear DNA fragmentation in the suspensor at the 16-celled embryo stage in the hybrid (SR1 × Hamayan). (**c**) Nuclear DNA fragmentation in the suspensor at the 32-celled embryo stage in the hybrid (Hamayan × SR1). (**d**) Nuclear DNA fragmentation in the suspensor at the 32-celled embryo stage in Hamayan. (**e**) Nuclear DNA fragmentation in the suspensor at the 32-celled embryo stage in SR1. (**f**) Nuclear DNA fragmentation in the suspensor at the 32-celled embryo stage in the hybrid (SR1 × Hamayan). (**g**) Nuclear DNA fragmentation in the suspensor at the small globular embryo stage in the hybrid (Hamayan × SR1). (**h**) Nuclear DNA fragmentation in the suspensor at the small globular embryo stage in Hamayan. Asterisks indicate the TUNEL-positive cells (**a**–**h**).

The maternal *NtCYS* allele was predominantly expressed in the reciprocal hybrids during early embryogenesis. These observations indicated that the initiation of PCD in the suspensor regulated by *NtCYS* was controlled by both parental transcripts during the early stage, and that maternal transcripts are expressed dominantly. Therefore, it is interesting to determine whether there are any distinctions in the initiation of PCD among SR1, Hamayan, and their hybrids. The time course and basic pattern for the initiation of PCD in the suspensor were compared between SR1 and Hamayan. Terminal deoxynucleotidyl transferase (TdT)-mediated dUTP nick-end labeling (TUNEL) as a marker of PCD showed that all cells in both the apical and basal cell lineages from the zygote to eight-celled embryo stage maintained nuclear DNA integrity. The first TUNEL signal was detected in the enlarged basal cell of the suspensor in 16-celled SR1 embryos and 32-celled Hamayan embryos. In both species, PCD accrued first in the lowest suspensor cell and later progressed upward to the uppermost cell. The TUNEL signal was first detected in the second suspensor cell from the basal end in 32-celled SR1 embryos, whereas it was detected much later in Hamayan at the small globular embryo stage. These results indicated that the basic process of PCD in the suspensor cell is the same in both species. PCD occurred obviously later and proceeded more slowly in Hamayan compared with SR1 (Fig. 4).

**Figure 5:**
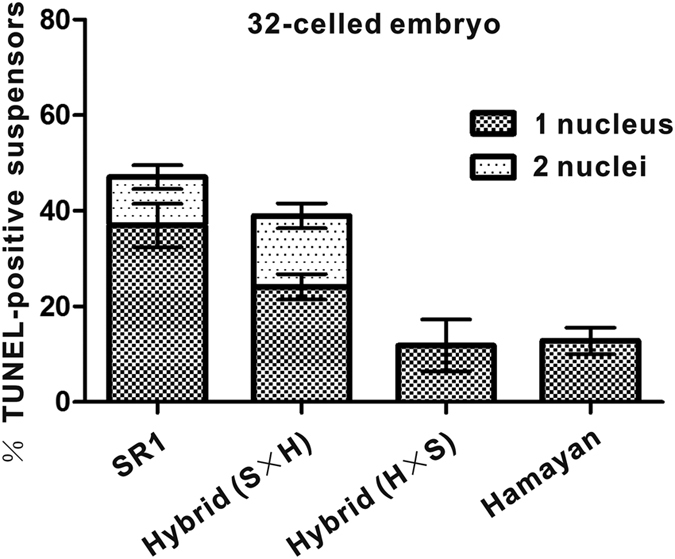
The frequency of suspensors containing indicated numbers of TUNEL-positive nuclei at the 32-celled embryo stage in SR1, hybrid (SR1 × Hamayan), hybrid (Hamayan × SR1) and Hamayan. Data represent the mean ± SE, n =  54–87.

TUNEL signals were also seen in the embryos of reciprocal crossed hybrids. The signal was maintained in the enlarged basal cell of the suspensor and then progressed acropetally to the uppermost suspensor cell, similar to that seen in their parents (Fig. 4). It seemed that the basic pattern of PCD initiation in the suspensor was conserved, and differences were only observed in the PCD time course. Interestingly, the hybrid embryos showed more maternal properties in the initiation and progression of PCD. The time course of PCD in the hybrids was almost the same as that in the maternal line (Fig. 5). These results also suggested that maternal *NtCYS* alleles mainly control the initiation of PCD in the suspensors of interspecific hybrids.

## Discussion

Maternal control during early embryogenesis in mammals is clearly evident^7^. However, in higher plants, both paternal and maternal transcripts contribute to embryogenesis during the early stages, although there is still debate regarding the contributions of each parental transcript^8^,^9^,^15^,^16^,^17^. Autran *et al*.^8^ reported that although maternal transcripts are predominant at the two- to four-celled proembryo stage, low levels of transcripts from the paternal genome only were already present. As embryogenesis progressed, the relative amounts of paternal transcripts increased at the globular embryo stage due to gradual activation of the paternal genome. Subsequently, Nodine *et al*.^9^ found that the overwhelming majority of transcripts from paternal and maternal alleles contributed equally, but a small fraction of transcripts derived from one parent make greater contributions during the initial stages of embryogenesis. Encouragingly, the first imprinted gene identified in plant embryos, *mee1*, was found to be expressed maternally in maize^18^, and more imprinted genes were later discovered in *Arabidopsis*^19^. In previous work, we confirmed marked differential contributions to zygote development from genes of parental origin in tobacco hybrid embryos^12^. The above results suggest that some specific developmental events during early embryogenesis are regulated by these genes of parental origin. Unfortunately, which genes of parental origin function in which specific developmental events remains unknown.

In our previous work on interspecies crosses, we noticed that the morphology of the hybrid suspensor is similar to its counterpart in the maternal parent. As the suspensor is a transient structure and is not involved in the heredity of any parent characters to the next generation, we speculated that suspensor development is regulated maternally. To test this proposal, NtCYS-regulated suspensor PCD as a suspensor-specific phenomenon is an ideal developmental event for careful observation. In contrast to our expectations, the initiation of PCD in the suspensor was shown here to involve both parental *NtCYS* transcripts in embryonic development of hybrid seeds from reciprocal cross between Hamayan and SR1. As confirmed in our experiments, both parental transcripts were detected in either two-celled proembryos or eight-celled embryos (Fig. 3).

However, as described above, although both parental transcripts are involved in the initiation of PCD in the suspensor, the expression level of the maternal *NtCYS* allele in hybrid embryos is much higher than that of the paternal allele, especially in two-celled proembryos. This observation further suggested that the paternal transcripts have less important roles, if any, in regulating initiation of PCD in the suspensor. Therefore, during the early embryonic stage, dominantly expressed maternal transcripts may play a major role in regulating the initiation of PCD. This result was consistent with the pattern of PCD in the hybrid suspensor, which was similar to that of the maternal line.

TUNEL staining was used to examine the dynamics of suspensor PCD during embryonic development in the basal cell lineages of proembryos. During early embryonic development, the TUNEL signal progressed gradually from the basal cell upward to the uppermost cell of the suspensor in both hybrid embryos and maternal embryos at a similar pace. Interestingly, the cell numbers of the suspensor in interspecific hybrids were also similar to those of the maternal lines, as shown in Fig. 1.

As it is difficult to identify specific SNPs in the *NtCYS* sequences between closely related species, Hamayan was selected from among more than 20 species or cultivars for use in this study. It is unclear whether there are any differences between interspecies and intraspecies crosses with regard to hybrid embryonic development. Fortunately, it is relatively easy to cross species of *Tabacum* and produce fertile hybrid plants. As shown in Supplementary Fig. S2, the embryogenesis of hybrids progressed normally as seen in their maternal lines. Especially, the initiation pattern of PCD in the suspensor detected in early embryos was consistent between maternal lines and their hybrids (Fig. 4). We did not observe any variations in the morphology or structure of the hybrid embryos, which may indicate the influence of interspecies hybridization during embryogenesis. Therefore, our observations regarding the initiation of PCD in the suspensor of interspecific hybrids between SR1 and Hamayan may reveal a general phenomenon.

A previous study indicated that all of the maternally imprinted genes in the *Arabidopsis* embryo are expressed in the seed coat, and some even showed slightly biased expression toward the basal embryo and the suspensor^19^. These observations indicate that many genes of maternal origin may be involved in development of the suspensor. The dominantly expressed maternal *NtCYS* transcripts regulating the initiation of PCD in the suspensor during the early stage of interspecific embryonic development provided a clear example of the involvement of genes of parental origin in early embryogenesis concerning a specific developmental event. Further studies evaluating a greater number of genes of parental origin are needed to elucidate the mechanism underlying the regulation of suspensor cell development.

## Materials and Methods

### Plant growth conditions

*Nicotiana tabacum* var. SR1 and *N. rustica* var. Hamayan were grown in a greenhouse at Wuhan University at 25 °C under a 16-hour light period.

### Interspecies hybridization

For the Hamayan × SR1 cross (the maternal parent is denoted first), mature SR1 pollen was pollinated directly on the stigma of Hamayan, which had been castrated previously^12^. For the SR1 × Hamayan cross, the SR1 styles of mature flowers were first shortened to suit the length of the Hamayan pollen tube. The petals were then removed, and the cut ends of SR1 styles were soon immersed in sterile pollen culture medium (20% sucrose, 0.01% H_3_BO_3_, 0.1 mM CaCl_2_, pH 5.6) for several minutes. Then, Hamayan pollen was placed on the cut ends of the SR1 styles. As the rate of fertilization was comparably low, 5 μg/ml 1-naphthaleneacetic acid was added at the joint of the ovary and the maternal tissue to avoid premature drop. The detailed procedure of the cross is illustrated in Supplementary Fig. S1. Expanded hybrid ovules were isolated from the ovaries at 5–7 DAP (days after pollination) for the experiments.

### Construction of cDNA pools from two-celled proembryos and eight-celled embryos

Embryos of SR1, Hamayan, and hybrids were isolated by a method described previously^12^,^13^,^14^. The isolated cells were transferred immediately to 2× lysis/binding buffer in 0.2-ml tubes, frozen in liquid nitrogen, and stored at –80 °C for subsequent mRNA isolation (Dynabeads mRNA DIRECT Micro Kit) and cDNA pool construction (SMARTer^®^ Ultra™ Low Input RNA Kit for Sequencing v3).

### Primer design for detection of *NtCYS* alleles

The nucleic acid sequence of *NtCYS* in SR1 has been reported^11^, and primer pair 1 was designed based on the sequences at each end of the gene (sense: 5′-ATGGCTTTCAAAATCAATTCTTT-3′; antisense: 5′-AGCAAACTTTTCGAAGGAAATG-3′) (Fig. 2b). As two conserved regions without any SNPs were found in *NtCYS* homologs, primer pair 2 was designed based on these conserved sequences to allow amplification of genes according to their abundance in the template (sense: 5′-ATCTTTTGCCATGTTTCTGC-3′; antisense: 5′-TCGTTGACAACAGCCAAA -3′) (Fig. 2b).

### Determination of the relative expression levels of *NtCYS* alleles in reciprocal hybrids

Primer pair 2 was used to amplify the *NtCYS* alleles using cDNA from two-celled proembryos of the hybrid (Hamayan × SR1, the maternal parent is denoted first), and a single band was obtained, and 211 clones were sequenced. The SNPs in the alleles were used as markers to distinguish among them. The relative expression levels were determined by counting the frequencies of the *NtCYS* alleles. Similarly, the relative expression levels of *NtCYS* alleles were also determined in two-celled proembryos from the hybrid (SR1 × Hamayan) by sequencing 260 clones; in eight-celled embryos from the hybrid (Hamayan × SR1) by sequencing 210 clones; in eight-celled embryos from the hybrid (SR1 × Hamayan) by sequencing 396 clones.

### Cell number and DNA fragmentation analyses

For detection of cell numbers and nuclear DNA fragmentation, ovules were fixed in 4% paraformaldehyde, and embryos were isolated by the method described above. The embryos were manipulated using the DeadEnd™ Fluorometric TUNEL System (Promega), according to the manufacturer’s instructions, to observe DNA fragmentation. Cell numbers were counted by the observation of nuclei. The embryos were incubated in 10 mg/ml 4′, 6-diamidino-2-phenylindole in 11% mannitol for 15–20 minutes, followed by two washes with 11% mannitol.

### Statistical analysis

All data are presented as the mean and SE. Chi-square test was used for the statistical analysis of the data, and p values of <0.01 were considered as significant difference.

## Additional Information

**How to cite this article**: Luo, A. *et al*. Initiation of programmed cell death in the suspensor is predominantly regulated maternally in a tobacco hybrid. *Sci. Rep*. **6**, 29467; doi: 10.1038/srep29467 (2016).

## Supplementary Material

Supplementary Information
