# Supplementary material for: Initiation of programmed cell death in the suspensor is predominantly regulated maternally in a tobacco hybrid
Source: Sci Rep. 2016 Jul 19;6:29467. doi: 10.1038/srep29467 (PMC4949469; doi:10.1038/srep29467)
Supplement: Supplementary Information [file srep29467-s1.pdf]

Initiation of programmed cell death in the suspensor is predominantly regulated maternally in a tobacco hybrid

An Luo[1, 2], Peng Zhao, Li-Yao Zhang, Meng-Xiang Sun[1]*

Supplementary material includes two figures and one table.

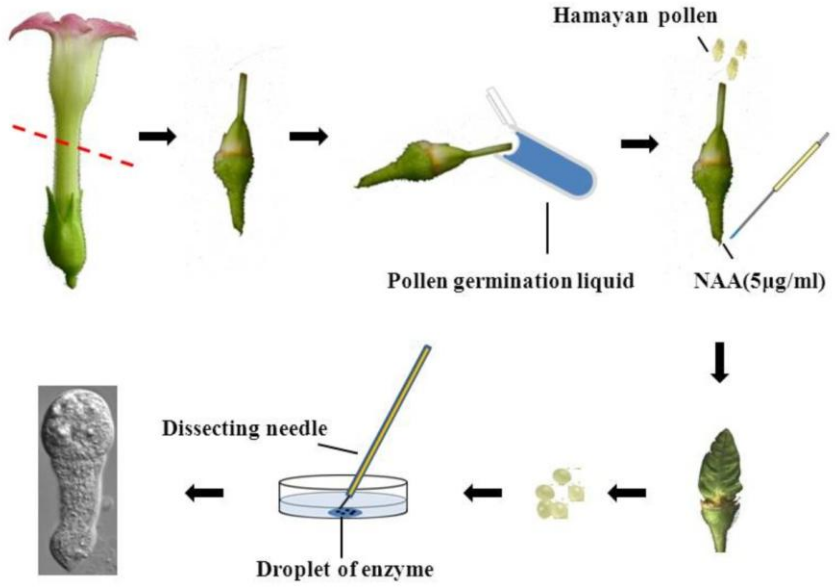

Figure S1. The procedure of obtaining of hybrid (SR1×Hamayan). Briefly, the SR1 styles of mature flowers were first shortened to suit the length of the Hamayan pollen tube by handmade razor. The petals were then removed, and the cut ends of SR1 styles were soon immersed in sterile pollen culture medium (20% sucrose, 0.01% H3BO3, 0.1 mM CaCl2, pH 5.6) for several minutes. Then, Hamayan pollen was placed on the cut ends of the SR1 styles. 5 μg/ml 1-naphthaleneacetic acid was added at the joint of the ovary and the maternal tissue to avoid premature drop. Expanded hybrid ovules were isolated from the ovaries at 5 – 7 DAP (days after pollination) for the experiments.

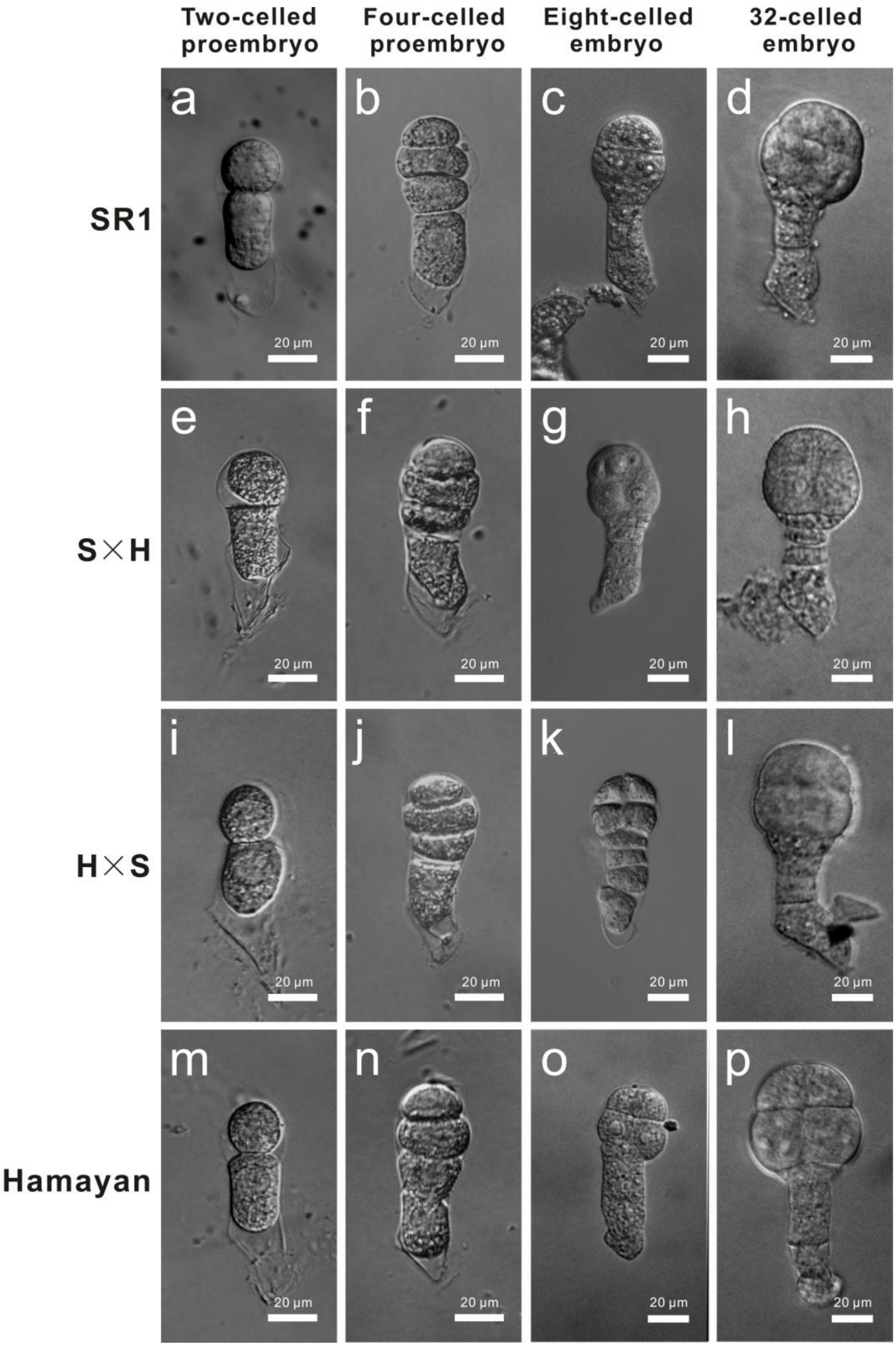

Figure S2. Early embryogenesis in tobacco SR1, Hamayan and their hybrids. (a-d): Isolated proembryos and embryos at different stages from SR1. (e-h): Isolated proembryos and embryos at different stages from the hybrid(S×H). (i-l): Isolated proembryos and embryos at different stages from the hybrid(H×S). (m-p): Isolated proembryos and embryos at different stages from Hamayan.

Table S1. Varieties of tobacco with no SNP at *NtCYS* alleles.

Varieties	Species
Southern Rhodesia	*Nicotiana tabacum*
Yunyan 87	*Nicotiana tabacum*
Bailei 21	*Nicotiana tabacum*
DW38	*Nicotiana tabacum*
Eyan	*Nicotiana tabacum*
LAB21	*Nicotiana tabacum*
Md609	*Nicotiana tabacum*
Md872	*Nicotiana tabacum*
Honghuadajinlong	*Nicotiana tabacum*
Long 2911	*Nicotiana tabacum*
KY14	*Nicotiana tabacum*
